# Subjective Versus Quantitative Methods of Assessing Breast Density

**DOI:** 10.3390/diagnostics10050331

**Published:** 2020-05-21

**Authors:** Wijdan Alomaim, Desiree O’Leary, John Ryan, Louise Rainford, Michael Evanoff, Shane Foley

**Affiliations:** 1 Radiography & Medical Imaging, Fatima College of Health Sciences, Abu Dhabi, UAE; 2 Radiography (Diagnostic Imaging), Keele University, Keele ST5 5BG, UK; D.s.o'leary@keele.ac.uk; 3 Radiography & Diagnostic Imaging, School of Medicine, University College Dublin, 4 Dublin, Ireland; John.ryan@ziltron.com (J.R.); Louise.rainford@ucd.ie (L.R.); Shane.foley@ucd.ie (S.F.); 4 American Board of Radiology, Tucson, AZ 85711, USA; Mevanoff@theabr.org

**Keywords:** breast density, breast imaging, quantitative density assessment, automated volumetric breast density measurement, VOLPARA, ImageJ, BI-RADS, American College of Radiology Breast Imaging Reporting and Data System, mammographic breast density

## Abstract

In order to find a consistent, simple and time-efficient method of assessing mammographic breast density (MBD), different methods of assessing density comparing subjective, quantitative, semi-subjective and semi-quantitative methods were investigated. Subjective MBD of anonymized mammographic cases (*n* = 250) from a national breast-screening programme was rated by 49 radiologists from two countries (UK and USA) who were voluntarily recruited. Quantitatively, three measurement methods, namely VOLPARA, Hand Delineation (HD) and ImageJ (IJ) were used to calculate breast density using the same set of cases, however, for VOLPARA only mammographic cases (*n* = 122) with full raw digital data were included. The agreement level between methods was analysed using weighted kappa test. Agreement between UK and USA radiologists and VOLPARA varied from moderate (κw = 0.589) to substantial (κw = 0.639), respectively. The levels of agreement between USA, UK radiologists, VOLPARA with IJ were substantial (κw = 0.752, 0.768, 0.603), and with HD the levels of agreement varied from moderate to substantial (κw = 0.632, 0.680, 0.597), respectively. This study found that there is variability between subjective and objective MBD assessment methods, internationally. These results will add to the evidence base, emphasising the need for consistent, simple and time-efficient MBD assessment methods. Additionally, the quickest method to assess density is the subjective assessment, followed by VOLPARA, which is compatible with a busy clinical setting. Moreover, the use of a more limited two-scale system improves agreement levels and could help minimise any potential country bias.

## 1. Introduction

Mammographic breast density (MBD) represents the amount of fat and fibroglandular tissue, which is a mixture of fibrous tissue, glandular tissue, collagen and epithelial cells that line the ducts of the breast [1,2]. Increasing MBD is thus associated with greater breast cancer incidence and is regarded as a risk factor for breast cancer [1,2]. Additionally, MBD has been associated with reduced sensitivity and thus decreased diagnostic accuracy in the detection of any lesions on mammographic images, which may lead to non-detectable cancers [3,4,5,6].

Several methods of breast density classification have been developed, both subjective and quantitative [1,7,8]. Studies using subjective BI-RADS assessment as used in clinical practice [9], or using Cumulus software, a visual thresholding technique [10]. 

Yaffe, in 2008, described several other quantitative methods to measure and classify breast density subjectively or objectively [1]. These include two-dimensional dense area measures, which do not incorporate the thickness of the dense area, Hand Delineation (HD) and semi-automated thresholding technique with ImageJ (IJ). One of these methods, HD, is still considered to be the gold standard in assessing breast density [11,12]. Li et al. developed IJ based on a public domain Java image-processing programme while imitating Cumulus HD [12]. Sovio et al., compared IJ and Cumulus, however, only MLO analogue images were used, left MLO for the Cumulus and both MLOs’ for IJ [13]. The study found that IJ is a valid method as a substitute for Cumulus (gold standard), with high inter and intra reader reliability in research settings [11]. However, this is not reproducible in clinical settings [14]. These methods are challenging in practice, tedious and time-consuming [15,16]. Furthermore, any misjudgment of the grey scale would lead to inaccurate breast density classification [16]. For this reason, Cumulus is only used as a gold standard in research and to create further quantitative methods for measuring breast density.

Alternatively, fully automated methods incorporate 3D measurements of dense areas, providing a more comprehensive assessment of the volume of dense breast tissue, which has been shown to be an indicator of increased risk of breast cancer [17,18,19].

Currently, there are a number of fully automated methods that quantitatively and objectively measure breast density [20,21]. One of the most well-known pieces of software is VOLPARA (Volpara Solutions, New Zealand), which has received Food and Drug Administration (FDA), Health Canada, the Therapeutic Goods Administration (TGA) approval, as well as the CE mark for use in mammography practices and has been used widely in clinical practice [22]. 

Many studies found an inter-rater variability between the subjective assessment and fully automated method in categorizing breast density [23,24,25,26]. However, to the knowledge of the authors no study has compared a larger number of radiologists across different regions assessing the same cases (including both right and left breast images) with VOLPARA, Cumulus HD and semi-objective IJ. Therefore, this study endeavours to address this deficiency, by examining the inter-rater variability between a range of methods currently used in both research and clinical settings worldwide in categorising breast density. This study uses a larger number of mammography cases and a large group of radiologists from two jurisdictions. This could provide a clear idea of the competence of this software globally.

Furthermore, Ko et al. proposed reducing the BI-RAD’S and VOLPARA classification scale from four categories to just two, in order to minimize variations when categorizing MBD [27]. The method was tested in this study with the aim of identifying a method that would be most consistent, simple and time-efficient in discriminating mammographic density, while simplifying the creation of an individual imaging pathway for different densities. 

Breast density notification legislation launched in many USA states from 2013 [28]. Additionally, many studies have proven that the inconsistency exists between radiologists globally when using subjective assessment [29,30]. This research was therefore timely in examining the efficiency of some of these objective and semi-objective methods compared to the subjective breast density evaluation, internationally. 

MBD outcomes assist in defining an individualized patient imaging pathway for follow up imaging with the least amount of radiation exposure for the radio-sensitive breast tissues. Additionally, MBD is important for each patient’s breast cancer risk. A consistent categorization system has the potential to help reduce potential variations in breast density assessment [31]. Such variations can lead to unnecessary imaging or biopsies and increased patient anxiety [31,32,33]. Therefore, the current study was conducted to compare mammographic density evaluation methods (Hand Delineation, ImageJ and VOLPARA) with the subjective assessment of USA and UK radiologists. Additionally, to our knowledge no previous study has looked at the impact of distractors within mammographic images in order to document which distractor has the greatest impact on radiologist BI-RADS decision making. According to Ko et al., both the asymmetry of breast size and lesions may cause disagreement between the MBD assessment methods [27]. Furthermore, the timing for each assessment method will be calculated to find the most sufficient and efficient method for clinical setting.

## 2. Materials and Methods 

An exemption from full ethical approval was granted by the University College Dublin, Human Research Ethics Committee (HREC). (Approval ID: LS-E-13-100-Alomaim-OLeary, May 15th 2013).

Permissions were granted by the American Board of Radiology (ABR), AZ, USA, for their examiners and by the British Society of Breast Radiology (BSBR), Hertfordshire, UK, for their respective member attendees to be both recruited voluntarily as participants to undertake this study. Both participants were recruited via local advertising. USA and UK participants’ years of experience reporting mammographic cases were recorded. Additionally, UK participants confirmed that they all were breast radiologists.

### 2.1. Mammographic Cases

Fully anonymised digital mammographic cases (*n* = 250), comprised 180 cases and 70 repeated cases. These cases were collected as part of previous research, from 18 centres in a national breast screening programme, with full patient consent. Each mammography case included “Right and Left; Mediolateral-Oblique and Cranial-Caudal”. The researcher (2+ years mammography experience) and a lead researcher and expert in mammography (5+ years), using consensus agreement along with the ACR-BI-RADS Atlas 4th edition selected and categorised the cases using BI-RADS. The cases were selected based on the following criteria: cases with the least artefacts, lesions causing deviation from the norm, technical errors and amount of asymmetry of breast size and MBD (left vs. right). However, one patient image per set did include a distractor for a challenge. The cases were randomly split up into five groups of 50 mammographic cases, including 36 cases and 14 repeated cases. 

For the purpose of avoiding radiologists’ sense of predictability, the density distribution was not equal within each set (Table 1). Additionally, 50 cases were considered sensible, time permitting, as the radiologists had the choice to review more than one set where possible. Furthermore, using R Package ‘KappaSize’, the power of the study was calculated to ensure it exceeds 80%. In accordance with previous studies, where a set of 30 mammographic cases reviewed with a minimum of three radiologists were defined as the minimum requirements to have a valid statistical analysis regarding inter-observer agreement level [34,35,36]. The sets of cases were presented using Ziltron software (Ziltron Ltd., Dublin, Ireland), which allows the cases to be viewed in high-quality, with easy navigation for users to move between cases, pan/zoom and alter the image contrast. Ziltron instruction was provided to both cohorts.

To facilitate the automated density analyser (VOLPARA, version 1.5.0, Volpara Solutions, Wellington, New Zealand) to automatically measure density, only those mammographic cases (*n* = 122) with full raw digital data were included [37]. The categorization breakdown of the dataset as reported by the researcher and VOLPARA are listed in Table 2 below. Additionally, for quality control purposes, the researchers’ preliminary BI-RADS categorisation data are tested with the three quantitative methods (VOLPARA, Hand Delineation and ImageJ), for consistency.

### 2.2. Subjective Density Assessment

The target population consisted of breast radiologists from both USA and UK who were purposively sampled at two educational events, following voluntary recruitment. Datasets were displayed on secondary reporting monitors using two computer screens, each one with 20” full viewable diagonal area, with landscape and portrait display modes, called ’ViewSonic ViewPanel, VP201mb (Viewsonic Corporation, Brea, CA, USA) with 1200 × 1600 pixel resolution for USA participants and a TOBII 23” TFT (TOBII Technology, Stockholm, Sweden) with 1920 × 1080 pixels screen resolution, for UK participants. The luminance level of each monitor for both cohorts was first checked using the Digital Imaging and Communications in Medicine (DICOM). Part 14: Grayscale Standard Display Function (GSDF) using VeriLUM calibration software and pod (ImageSmiths) to ensure all screens met the standard range [37,38]. The measurements for the USA first screen: 164.2 candela per square metre (cd/m^2^) maximum, 0.35 cd/m^2^ minimum and for the second screen: (175.5 cd/m^2^ max, 0.31 cd/m^2^ min), for UK screen: (300 cd/m^2^ max, 0.67 cd/m^2^ min).

All participants were asked to simply record their subjective MBD score using the Breast Imaging Reporting and Data System (BI-RADS) 4th edition (almost fatty, scattered fibroglandular densities, heterogeneously dense and extremely dense) [9], for each image displayed. 

### 2.3. Quantitative Assessment

Byng et al., [39] and Boyd et al., [40], both used 6 MBD categorisation (0%, 1% to <10%, 10% to <25%, 25% to <50%, 50% to <75%, and ≥75%), but later work condensed this to four by combining the upper two categories (<10%, 10–24%, 25–49% and ≥50%.) [13], as a consequence of the small number of subjects in some mammographic density categories. Also ≥50% of mammographic density are counted as dense breast [41]. The modified four category approach was used in this study for the semi-objective (IJ) and semi-subjective (HD) assessment of the MBD.

#### 2.3.1. Semi-Subjective Density Assessment

**Figure 1:**
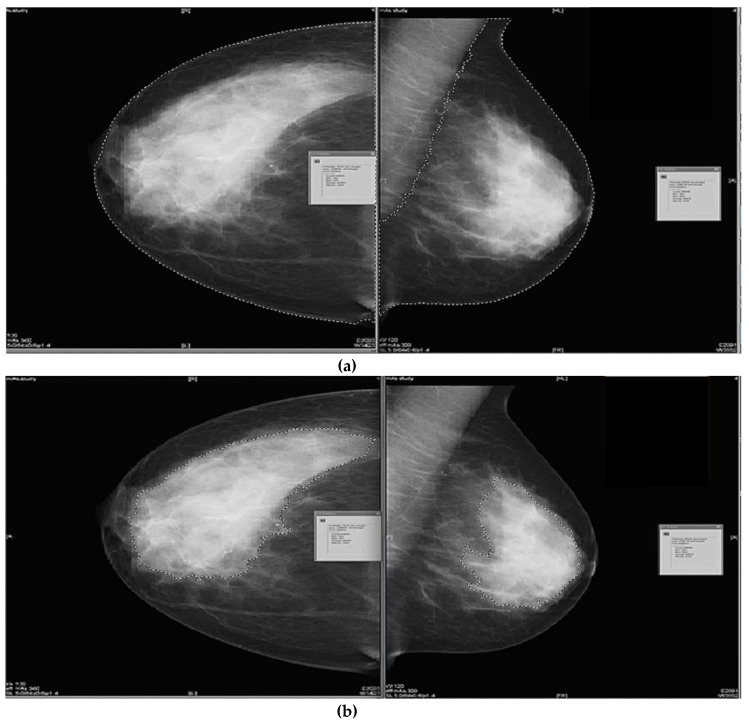
(**a**). Screen shot of the semi-subjective (HD) method showing the craniocaudal and mediolateral oblique breast images with boundaries drawn around the outer surface of the breast. (**b**). Screen shot of the semi-subjective (HD) software showing the craniocaudal and mediolateral oblique breast images with boundaries drawn around the fibroglandular tissue within the breast.

Using GE Centricity RA 600 (GE Healthcare, Milwaukee, WI, USA) image software. The images were displayed on two side-by-side Barco (Barco NV, Belgium) Coronis 3 Mega Pixel (MFGD-3420) screens, with luminance levels of 402.6 cd/m^2^ max, 0.41 cd/m^2^ min and 529.1 cd/m^2^ max, 0.63 cd/m^2^ min respectively. The area of the breast of each mammographic image for each projection (RCC, RMLO, LCC, LMLO) was quantified by selecting the contrast and density toolbar to adjust the contrast and density to enhance the skin edge of the breast. Then, the freehand area calculation tool was selected from the menu, to draw a semi-circular boundary around the area of the breast using a computer mouse pad and polygon generating a measurement in mm^2^. The measurements of the delineation of the breast area for the MLO images were done by tracing individually around the area of the whole breast excluding the pectoral muscle, see Figure 1a [16].

To measure dense breast tissue, the same steps were followed, by adjusting the contrast and density to enhance the fibroglandular tissue and using a computer mouse pad and polygon to draw around the area of interest. The measurement was generated in mm^2^, see Figure 1b.

Both area measurements were converted to cm^2^ then multiplied by the depth of the breast on the mammogram image in order to calculate the whole volume of breast tissue versus the volume of dense breast tissue. The ratio of the density was thus calculated in a semi-subjective manner. 

#### 2.3.2. Semi-Objective Density Assessment

**Figure 2:**
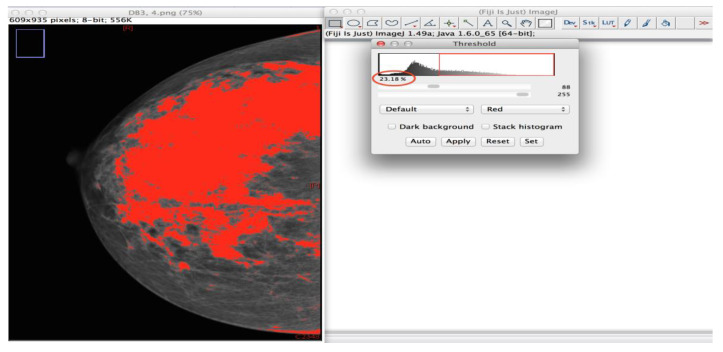
Screenshot of the semi-objective (ImageJ) software, showing a right Cranio-Caudal image with the density as highlighted and the red circle represents the volume density percentage by the software.

This assessment method uses an automated thresholding procedure that separates the interest area (dense tissue) from the background. The dense portion of the breast was segmented via IJ freeware software for Portable Network Graphics images, which is a built-in design extended by Fiji plugin a 3rd party Java modules [42]. This software performed an area measurement, which it then represented as a percentage. This software also allows the user to alter the area selected by the software. The software performed the area measurement using the interfaces/pixel edges between the various portions of dense tissue in the breast, thus the proportion of dense breast to fatty breast tissue was calculated (Figure 2). For more accurate measurement, the pectoral muscle was cropped manually from the MLO images [10,43].

#### 2.3.3. Automated Objective Density Assessment

**Figure 3:**
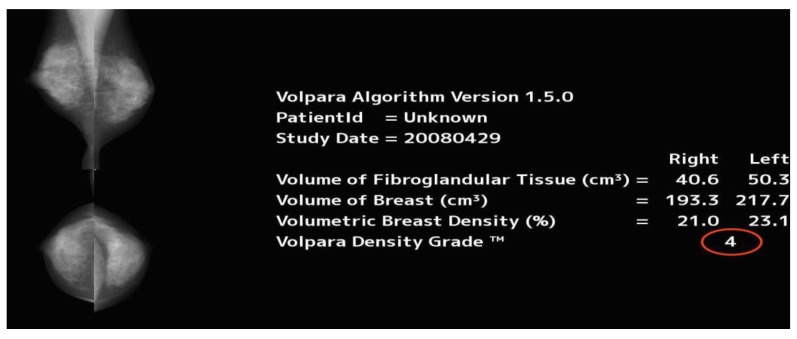
Screen shot of an example of the automated objective density assessment (VOLPARA) software; the red circle shows the BI-RADS as graded by the software.

The test set of patient digital mammography cases were saved as raw data DICOM images and then uploaded into a fully automated volumetric analysis, VOLPARA (version 1.5.0, Volpara Solutions, Wellington, New Zealand) density assessment software for quantitative assessment of MBD. Image analysis begins by locating a reference point in the breast, mainly close to the chest wall which contains fatty tissue. Following that, the x-ray attenuation is calculated for each pixel. A density map is created by calculating the attenuation degree in a pixel, the x-ray source and the tissue composition located between the pixel. VOLPARA computes the volume of the fibroglandular tissue and the breast (both in cm^3^), volumetric MBD (%), all by analysing the values in the density map. Then the volumetric density is calculated from these data, ranging from 0% to 40%. The information from MLO and CC images are averaged, and then the density information is given per breast. Following that, the density category is provided for each patient. To obtain percentage density, total fibroglandular tissue volume is divided by total breast volume. The percentage density is graded as following: BI-RADS1 (<4.5%), BI-RADS 2 (≥4.5% and <7.5%), BI-RADS 3 (≥7.5% and <15.5%) and BI-RADS 4 (≥15.5%) [44]. The final step automatically processes the data and sends a DICOM secondary capture image to the radiologist’s screen, see Figure 3 [45]. The MBD score is given according to the BI-RADS MBD classifications [46,47], which allows for comparison with other methods used in this study. 

### 2.4. Time Difference between Methods

The average length of time for HD, IJ and VOLPARA was measured using a digital stopwatch (iPhone 6 plus, Apple Inc., California). A random stratified sample of eight cases (two cases from each BI-RADS category) was selected for both the HD and IJ methods. Meanwhile, the time taken by VOLPARA to provide the BI-RADS for nine cases saved on one compact disc (CD) was measured. Finally, the length of time for the subjective BI-RADS categorization was calculated using ZILTRON software instant reporting and feedback of the length of time taken by each radiologist to move from one image to the next image for the five sets of cases. In addition, the time was calculated individually for the cases with and without distractors, as reported by the UK radiologists.

For the HD method: the average time was calculated for all BI-RADS except for the two BI-RADS 1 cases, as they were fatty breasts. This time was calculated by adding the drawing time taken for each image for the outer surface to the time taken for outlining the dense area. Times were taken for each of the four projections for each patient, and the average time was calculated.

### 2.5. Amended, BI-RADS Scale

To investigate further possible adaptations to the existing BI-RADS method, subjective BI-RADS ratings from radiologists in each country (USA and UK) and objective VOLPARA grades were additionally amended using just two categories (high vs. low). This was achieved by combining BI-RADS 1 and 2 together (Low Density), with BI-RADS 3 and 4 (High Density) as a second category.

### 2.6. Distractors within Cases That Impacted the Radiologists’ Breast Density Decisions

Further analysis was performed on the data collected for the cases with distractors from both cohorts. However, the USA radiologists, unlike the UK radiologists, rarely used or commented on this question; this will be explored in the discussion. Each answer from UK radiologists was counted and datasets created by the number of radiologists, thereby representing opinions from 1350 reads. An example is set C, which contains 50 cases read by six radiologists, yielding 300 reads. This procedure was used with all datasets.

Additionally, further analysis was performed to explore whether cases with distractors (*n* = 56) and cases without distractors (*n* = 66) would have an influence on the level of agreement between the subjective BI-RADS decision of UK radiologists and the automated software (VOLPARA) categorisation.

### 2.7. Data Analysis

The levels of agreement between the radiologists from both countries (USA and UK) assessing MBD were compared separately to VOLPARA using Weighted Kappa test (κw) (95% confidence interval) for the categorical items [48]. Additionally, the levels of agreement between all the different methods were compared using a Weighted Kappa test (κw) (95% confidence interval).

Intra-class Correlation Coefficient (ICC) (95% confidence interval) [49,50] was carried out to determine the intra-rater reliability for each radiologist, who completed more than one set of cases for the repeated cases within the sets.

The Spearman Correlation Coefficient (r) was calculated following the Li et al., [12] study methodology to test for an association between the HD, and IJ MBD measurements.

Kruskal–Wallis non-parametric ANOVA (Chi-Square Test) was used to determine whether there is a significant difference between the time taken for each mammographic density assessment method, including UK radiologists’ and VOLPARA [51,52].

## 3. Results

A total of 25 USA breast imaging expert radiologists with >8 years’ experience, post radiology board certification, as well as 24 UK breast imaging experts, participated, with half of the cohort having >8 years’ experience and only 12% having ≤1 years’ experience post radiology board certification.

### 3.1. MBD Assessments

Table 3 below shows that the levels of agreement between USA and UK radiologists with the fully automated method (VOLPARA), were substantial and moderate, respectively. Moreover, the table shows that the levels of agreement between USA and UK radiologists with semi-objective (IJ) and semi-subjective (HD) methods varied from moderate to substantial agreement.

Table 4 below shows the levels of agreement of VOLPARA with semi-objective (IJ) and semi-subjective (HD) methods varied from moderate to substantial agreement.

The intra-class correlation coefficient agreement for intra-rater reliability for the radiologists in both countries on the repeated cases within all five sets was high (ICC > 0.9): the average ICC measure for USA radiologists being 0.973 with a 95% confidence interval from 0.966 to 0.978 (F (219,876) = 36.974, *p* < 0.001). For the UK radiologists the average ICC measure was 0.927 with a 95% confidence interval from 0.821 to 0.975 (F (13,26) = 15.194, *p* < 0.001).

The findings for the quality control tests are: the level of agreement between the researcher’s subjective BI-RADS categorisation and VOLPARA and Hand Delineation was substantial (κw = 0.660 and 0.782, respectively, *p* < 0.001), while, with ImageJ was almost perfect agreement (κw = 0.856, *p* < 0.001).

The level of agreement using weighted Kappa between Hand Delineation and ImageJ, is substantial with κw = 0.699, (*p* < 0.001). 

When the correlation was tested between ImageJ and Hand Delineation assessment for breast density, a strong, positive association between the two subjects (*r* = 0.809, *p* < 0.001, 2-sided), was found.

Using the modified two grade scale the agreement levels increased to substantial agreement between VOLPARA and USA radiologists (0.702, *p* < 0.001) and UK radiologists (0.630, *p* < 0.001).

### 3.2. Time Analysis

Examination of the time difference between each method showed that there was a significant difference between each method (*p* < 0.001), with visual assessment of the MBD taking an average of 14 s (range: 7–33 s), VOLPARA takes approximately 57 sec for each patient, with HD and IJ methods taking an average time of 320 sec (range: 198–453 s) and 131 sec (range: 83–168 s), respectively.

### 3.3. Image Distractors Influencing MBD Assessment

**Figure 4:**
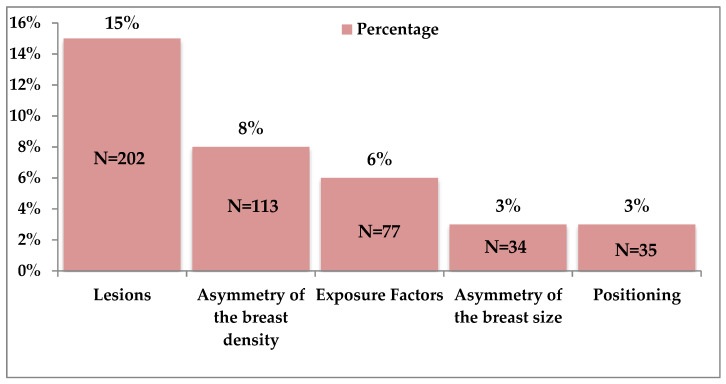
Percentage of the distractors within total cases according to the number of reads, as decided by the UK radiologists.

Of the original total of 250 mammographic cases, 92 mammographic cases with distractors were included. However, results show that different decisions were taken by each radiologist (*n* = 24), according to the number of reads. Figure 4 below shows that the highest percentage of distractors identified by radiologists while assessing breast density was lesions.

When the agreement level for the BI-RADS categorization between UK radiologists and VOLPARA for cases with distractors was calculated, the agreement was substantial (κw = 0.656, *p* < 0.001), meanwhile, the agreement level for the cases without any distractors was moderate (κw = 0.518, *p* < 0.001). The average time for the subjective categorization for cases with distractors was 12 s (range: 7–23), and 16 s (range: 9–33 s) for those without any distractors, however, the time difference was not significant (*p* > 0.05). Furthermore, VOLPARA takes approximately 57 s for each patient, whether the image is with or without any distractors.

**Figure 5:**
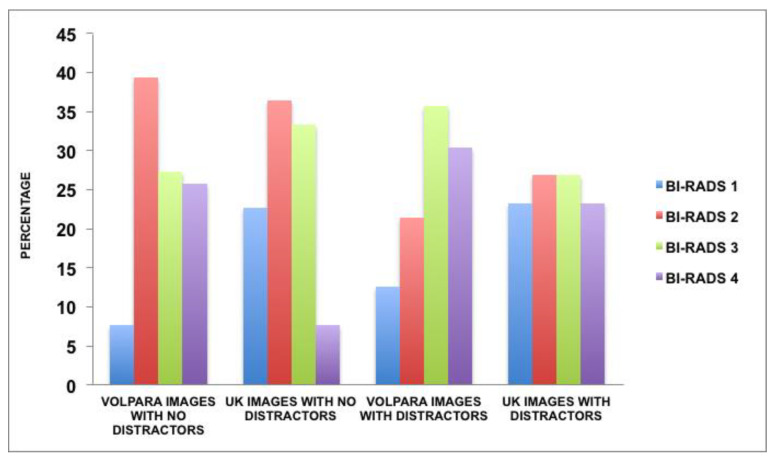
Distribution of the BI-RADS scoring amongst cases with and without distractors for VOLPARA and UK radiologists.

Figure 5 shows that for cases with no distractors, VOLPARA placed less cases in BI-RADS 1, while UK radiologists placed less cases in the BI-RADS 4. For the cases with distractors, VOLPARA placed most of the cases on the higher categories. However, UK radiologists placed cases more evenly across the categories.

## 4. Discussion

As MBD categorisation is subjective, this may lead to under- or overestimates of the percentage of fibroglandular tissue within the mammographic cases. This finding has been confirmed and it has been proposed that MBD should be assessed using automated software with a view to unifying MBD judgment worldwide [29,31,32,53], as the significance of proper MBD categorization is well documented [17,18,19].

When the radiology cohort findings were compared with the fully automated objective method (VOLPARA), it was found that this method has a substantial agreement with the American radiologists. However, a moderate agreement was found with the British radiologists. The higher agreement between USA radiologists and VOLPARA might be expected, as VOLPARA software thresholds were determined by a USA expert group of radiologists [54]. This difference between both cohorts and VOLPARA raises some concerns, as it may impact negatively upon patient imaging and follow up pathway [31,32,33,55].

Lee et al., [24], a Korean-based study, reported a substantial level of agreement (0.799) between a single radiologist and VOLPARA. However, another Korean-based study, found moderate agreement (0.54) between two radiologists and VOLPARA [17], while an Indian-based study comparing two radiologists’ BI-RADS assessment results with VOLPARA found fair agreement (0.398 and 0.388) [45]. Varying levels of agreement have thus been found internationally, especially where small cohorts of radiologists were involved. By contrast, in this work a large cohort from two countries participated, with the results varying again (from 0.589 to 0.639). This could be driven by the difference in experience levels, clinical practice and legal requirement to report MBD between the two cohorts. This difference may also be because UK radiologists are using a three-category system, meanwhile, the USA uses the four BI-RADS. Moreover, the viewing environments differed between the two cohorts, specifically the setting lighting and the display monitors [29]. However, both monitors were similar in quality and it was unlikely to influence these results [29].

Sartor et al., [56] in his study has explained a few reasons for the low agreement between VOLPARA and subjective BI-RADS categorization: BI-RADS are judged according to processed images, while, VOLPARA only analyses raw data which contains information about the pixel intensities and the X-ray attenuations of the fatty tissues versus the fibroglandular tissues, which calculate the depth of the fibroglandular tissues [57]. Moreover, as outlined, VOLPARA’s assessment scale is a continuous one, while, BI-RADS categories are an estimation that are divided into four groups, for this reason the MBD that is near the limits in the different VOLPARA’s assessment scales could be classified into the upper or the lower adjacent BI-RADS category, since the radiologists would not be able to detect small differences in mammographic density. Similarly, van der Waal et al. [58] state, following the direction of the ACR, that radiologists tend to rate at the maximum level, in contrast to VOLPARA, which is designed to estimate density using the average of multiple views.

Subjective variations noted worldwide could lead to increases in both radiologist and patient uncertainty. Radiologists may fear over or underestimating MBD, while patients may fear the possibility of a cancer being missed. Such variation could also create difficulties in following a consolidated MBD categorization, thus making it more difficult to develop a specific imaging pathway, which is dependent on MBD, and unifying patient care [32,55,59,60,61]. Additionally, it increases the woman’s concern of going through demanding procedures, the pain involved with extra biopsies and imaging and, more importantly, questions the reliability of mammography [31,32,33,55]. However, it is important to consider that the results of this study were collected without VOLPARA guidance to assist radiologists to categorize MBD. Therefore, the use of VOLPARA guidance is recommended. An American-based study of Schilling et al. [25] compared eight radiologists before and after using the guidance of VOLPARA. This showed that the level of agreement before the guidance of VOLPARA was moderate (0.566), but, following the aid of VOLPARA to re-categorize the same cases, the level of agreement became substantial (0.626). This is similar to the level of agreement between the American radiologists and VOLPARA in this study (0.639), where radiologists were not provided with VOLPARA’s decisions in advance, however they were ABR examiners, which means that they had more experience and training when compared to others. Meanwhile, the initial result before the aid of VOLPARA is similar to the level of agreement of the UK radiologists in the current study, where the radiologists possibly had less experience with the ACR BI-RADS and were not practicing MBD assessment in their routine practice. Moreover, Jeffers et al.’s [62] study found that the level of agreement between the subjective single image reader and VOLPARA was fair, however, this low level of agreement may be because the image reader only had two years of experience and categorised without the aid of VOLPARA. To elaborate further on this point using quality control test findings, the researcher’s level of agreement with VOLPARA was tested, and it was found to be substantial (0.660, *p* < 0.001), which was similar to the USA radiologists. This may be because the researcher has been specifically trained to use BI-RADS, and categorised the cases with the aid of BI-RADS Atlas 4th edition. These results add to the evidence supporting the fact that, without the assistance of VOLPARA, the image readers will have a lower agreement. This emphasises the value of incorporating VOLPARA or other such similar automated systems into MBD assessment as they may positively impact on the radiologists’ agreement, as also proven by Schilling et al. [25]. Thus, this current study recommends a further investigation between the radiologists and VOLPARA agreement levels when VOLPARA is used as a reference.

A further part of the study tested the agreement between the subjective analysis and VOLPARA on a more limited version of the current BI-RAD’S classification by reducing the scale to just two separate categories, to see whether differences in MBD interpretation could be reduced. The findings demonstrate that the level of agreement between the two cohorts’ subjective evaluation and VOLPARA showed more substantial agreement. Thus, the scale of two separate categories—low density and high density—is reliable and robust, and therefore could lead to reducing patient uncertainty, as well as potentially minimizing the costs associated with unnecessary imaging and additional procedures, and help in unifying the imaging pathway for patient cohorts.

Moving toward the area-based methods (HD and IJ), which assess MBD by segmenting two-dimensional mammographic images into dense and non-dense areas, and have been only partially automated [10,63], when the correlation between these two methods was tested, a high correlation was observed (*r* = 0.809). This was expected and is in agreement with previous studies of Li et al., [12] and Couwenberg et al., [64], as IJ was developed mimicking the HD method. Furthermore, using quality control findings, the level of agreement between HD and IJ and the researcher’s categorization according to the BI-RADS 4th ed, varied from substantial to almost perfect (0.782 and 0.856). Both methods were performed by the researcher. Meanwhile, the level of agreement between the two cohorts with HD and IJ was lower than for the researcher. These results support the assumption that these two methods are dependent on visual decisions to determine the breast area and the MBD edges. Furthermore, the intra-rater reliability for both UK and USA radiologists are in almost perfect agreement, which supports the possibility that individual categorization standards for each radiologist is strong and is one of the limitations that might affect final outcome. However, the researcher acknowledges that, in other studies, HD and IJ methods were performed by radiologists, rather than the researcher.

The second limitation, as stated by Byng et al. [10], is that the HD takes a greater amount of time, even when carried out by skilled and professional radiologists. Byng et al., [10] reported that the time to conduct the HD method is less than four minutes to evaluate the four mammographic images for each patient [10,16,65]. Meanwhile, in this study the HD took almost 5 min, and IJ almost 2 min to evaluate the four mammographic images, which, for HD, is greater than the time reported by Byng et al., [10], proving that this method is largely dependent on the subjective assessment of the image readers, as well as the skill level and training in the use of the method.

On the other hand, when the level of agreements between HD and IJ MBD assessment methods with VOLPARA were tested, moderate agreement was found between VOLPARA and HD (0.597) and IJ (0.603). The variation in agreement between VOLPARA and both HD and IJ is due to the fact that VOLPARA is a fully volumetric measurement, while both HD and IJ are area-based methods with visual computer assistance. The differences in the methodologies may have an impact on the final results, as area-based methods are highly user-dependent. The two-dimensional image of the compressed breast is segmented and the threshold is defined by the user. This increases the possibility of inter-observer variability [10,16,63,65]. In addition, according to van Engeland et al. [66] and McCormack et al.’s [67] studies, the potential source of variation between both methods is that, as a quantitative measure of MBD volume, fully volumetric methods are a more suitable approach than a projected area.

When agreements between HD and IJ breast density assessment methods with the BI-RADS subjective assessment were tested, the level of agreement between these two methods and the two cohorts was substantial. In general, this is higher than the agreement level between VOLPARA and the two cohorts. Possible explanations for this higher agreement include the fact that, as in volumetric methods, it is difficult to locate a reference point in the breast that only contains fatty tissues, either within an extremely dense breast or in a very small breast [66]. This software is also affected by asymmetry or asymmetric breast thickness as the breast thickness influences both breast and density volume; when the thickness increases, both volumes will increase [54]. Moreover, as the x-ray device nearly always underestimates breast thickness, VOLPARA’s assessment will increase, therefore, women will be placed in a higher BI-RADS category [54].

British radiologists when asked regarding potential distractors, reported lesions causing deviation from the norm as the greatest distractor, followed by the asymmetry of the MBD. Lee et al., [24] found that the asymmetry of bilateral MBD had the biggest impact on the disagreement between the BI-RADS assessment by the radiologists and VOLPARA. The level of agreement between the radiologists and VOLPARA for the cases with distractors, in this study, showed that the agreement level (0.451, *p* < 0.001) was higher than the agreement for the cases with no distractors (0.293, *p* < 0.001), which was not expected, and this supports the need for further studies to explore visual processing reasoning. Furthermore, as shown in Figure 5, the distribution of the BI-RADS differs between VOLPARA and UK radiologists. This may be the reason for the low agreement level. Additionally, high body mass index (BMI) is significantly related to volumetric-assessed MBD [68]. Another explanation is that, when the average viewing time was calculated for the radiologists viewing the cases with distractors compared to the cases without distractors, it was found that they spent less time on the cases with distractors than the cases without any distractors. This was not expected as, logically, the cases with possible distractors need more time to be explored, because the radiologists will have to focus on the general image plus the distractor. As has been stated by Eagleman et al. [69], perception time is still shrouded in mystery. As stated by Nodine et al. [70], the image reporter’s confidence level decreases when the interpretation time increases, which increases the likelihood of errors. In view of this research, further studies should be carried out to compare the level of agreement between radiologists with the time spent viewing cases, both with and without distractors. This could further test the hypothesis that radiologists viewing cases for longer times tended to both incorrectly diagnose and to misinterpret MBD.

The authors acknowledge that this work has some limitations: the two-dimensional nature of IJ and HD methods does not take into consideration the thickness of dense tissue within the breast [37,71]. This method is also a user-assisted (semi-automated) method, so it is only for research settings and not suitable for use in clinical settings [71]. The radiologists from UK and USA were asked to give their opinion as to whether there were any distractors within the image that might affect their assessment of MBD. Unlike their UK colleagues, the USA radiologists rarely used or commented on this question. These differences in response could be due to the time constraints, also this question was mandatory for the UK radiologists, as explained previously. Additionally, the use of repeated cases could be a limitation, by creating a memory effect in the rating of MBD by radiologists.The viewing environments and the monitors used differed for both cohorts, which was not ideal [29]. However, as observers were not seeking any abnormality per se and only categorizing overall density, these factors are thought not to be as marked or noticeable as if they were asked to look for any lesions within the breast, as density depends on the general overall view of the amount of fibroglandular tissue compared to the fatty tissue.

## 5. Conclusions

There is variability between breast density assessment methods, and that all methods are not in complete or even high agreement with each other. The level of agreement varied from moderate to substantial between VOLPARA with both cohorts. Despite the high agreement between HD, IJ and both cohorts, HD, IJ are user dependent, requires skills, training and are time consuming. Therefore, these are costly in relation to radiologists’ time and are impractical in a clinical setting. Moreover, subjective assessment is the quickest method to assess density, followed by VOLPARA, and both are appropriate in a busy clinical setting. In addition, the use of the limited two-scale system improves agreement levels, and thus could help in minimizing any potential country bias while providing a clear imaging pathway for patients. To date, there is no universal agreement as to which methods are best-suited to creating individual imaging pathways, therefore, further investigation is strongly recommended.

## Figures and Tables

**Table 1:** The distribution of BI-RADS categories within each set without the repeated cases and for the repeated cases.

Breast Density	Image Sets					
	A	B	C	D	E	Repeated Cases
BI-RADS 1	14%	25%	22%	17%	22%	7%
BI-RADS 2	36%	17%	22%	28%	25%	57%
BI-RADS 3	36%	39%	39%	39%	36%	21%
BI-RADS 4	14%	19%	17%	17%	17%	14%

**Table 2:** The distribution of BI-RADS categories for the 122 cases according to the researcher and VOLPARA.

Breast Density	Researcher	VOLPARA
BI-RADS 1	16%	10%
BI-RADS 2	29%	31%
BI-RADS 3	41%	31%
BI-RADS 4	14%	28%

**Table 3:** The level of agreement using (κw) between USA and UK radiologists with Hand Delineation, ImageJ, and VOLPARA.

Subjects	VOLPARA	Hand Delineation	ImageJ
USA radiologists	0.639 *	0.632 *	0.752 *
UK radiologists	0.589 *	0.680 *	0.768 *

* Statistical significance (*p* < 0.001).

**Table 4:** The level of agreement using (κw) between Hand Delineation and ImageJ, with VOLPARA.

Subjects	VOLPARA
Hand Delineation	0.597 *
ImageJ	0.603 *

* Statistical significance (*p* < 0.001).
